# 

**Published:** 1899-07

**Authors:** 


### THE NATIONAL ASSOCIATION OF DENTAL FACULTIES.

THE annual meeting of the National Association of Dental Faculties will be held at Niagara Falls, beginning July 28, at 10 A.M., 1899, and will continue until the 31st.

The Executive Committee of this body will meet on Thursday, the 27th, at ten o'clock, for the preparation of business.

This meeting will be an important one, and it is essential that all schools should be represented.

